# Expression TGM2 and BNIP3 have prognostic significance in laryngeal cancer patients receiving surgery and postoperative radiotherapy: A retrospective study

**DOI:** 10.1186/1479-5876-10-64

**Published:** 2012-03-30

**Authors:** Ting Jin, Huan-Xin Lin, Hui Lin, Li-Bing Guo, Nan Ge, Xiu-Yu Cai, Rui Sun, Wen-Kuan Chen, Qiu-Li Li, Wei-Han Hu

**Affiliations:** 1 Department of Radiation Oncology, Zhejiang Cancer Hospital, Hangzhou, Zhejiang 310022, People's Republic of China; 2 State Key Laboratory of Oncology in South China, Sun Yat -sen University Cancer center, Guangzhou, Guangdong 510060, People's Republic of China; 3 Department of Radiation Oncology, Sun Yat -sen University Cancer center, Guangzhou, Guangdong 510060, People's Republic of China; 4 Department of Oncology, Guangdong Second Provincial People's Hospital, Guangzhou, Guangdong 510060, People's Republic of China; 5 Department of Head and Neck Surgery, Sun Yat -sen University Cancer Center, Guangzhou, Guangdong 510060, People's Republic of China

**Keywords:** TGM2, BNIP3, Postoperative radiotherapy, Laryngeal cancer, Prognosis

## Abstract

**Background:**

This study was designed to determine the pattern and correlation between expression of the HIF-1α transcriptional targets TGM2 and BNIP3 in laryngeal cancer, and investigate the association of BNIP3 and TGM2 with clinical outcome in laryngeal squamous cell carcinoma (SCC) patients receiving postoperative radiotherapy.

**Methods:**

Immunostaining with antibodies specific to BNIP3 and TGM2 was performed in formalin-fixed, paraffin-embedded specimens from 148 laryngeal SCC patients. BNIP3 and TGM2 expression was scored as high or low, based on the number of tumor cells stained and the staining intensity. All patients received postoperative radiotherapy. Patient follow up and clinicopathological data were compared using the Chi-squared test, univariate and multivariate analyses, and survival curves were generated using the Kaplan-Meier method and log-rank test.

**Results:**

The 3, 5 and 10-year overall survival rates (OS) for all patients were 77.7%, 71.6%, 56.4%, respectively. Primary tumor site, T stage, overall stage, lymph-node metastasis, BNIP3 expression and TGM2 expression were significant prognostic factors for OS in univariate analysis. Negative cervical lymph nodes, high BNIP3 expression and low TGM2 expression were independent prognostic factors of improved OS in multivariate analysis. BNIP3 expression correlates with TGM2 expression in laryngeal SCC (*P *= 0.012).

**Conclusions:**

This study indicates that lymph-node metastasis, BNIP3 expression and TGM2 expression are independent prognostic factors in laryngeal SCC patients receiving postoperative radiotherapy. Further studies are required to investigate how BNIP3 and/or TGM2 influence the prognosis of laryngeal SCC patients treated with postoperative radiotherapy, and to determine how TGM2 and BNIP3 expression are regulated.

## Background

In the United States, laryngeal cancer accounted for approximately 0.85% of new cancer diagnoses and 0.65% of all cancer deaths in 2008 [1]. Postoperative radiotherapy (PRT) is widely advocated for squamous cell carcinoma (SCC) of the head and neck patients with a high risk of recurrence after surgical resection.

The most important factor in the prognostic evaluation of head and neck squamous cell carcinoma (HNSCC) is the tumor node metastasis (TNM) staging system, of which nodal stage is the most relevant factor. However, the outcome of patients with the same TNM stage can vary, which has led to a concerted effort to define additional TNM subcategories with a similar prognosis, and recent research has focused on the identification of molecular and biologic prognostic factors, regardless of TNM staging.

The hypoxic fraction of human cancers is resistant to radiation therapy due to reduced generation of oxygen radicals. The transcription factor hypoxia-inducible factor-1 (HIF-1α) upregulates expression of a variety of target genes under hypoxic conditions, and plays a major role in determining tumor radiosensitivity. Therefore, HIF-1α and HIF-1α target genes represent potential therapeutic targets to influence the effect of hypoxia on tumor radiosensitivity.

*BNIP3*, a hypoxia-inducible pro-apoptotic gene belonging to the BCL2 family, was originally identified as an adenovirus E1B19-kDa protein-interacting gene. In normal tissues, BNIP3 expression is upregulated in hypoxic conditions by hypoxia inducible factor HIF-1α and can lead to cell death [2-4]. In tumors, *BNIP3 *is silenced via epigenetic mechanisms, such as promoter hypermethylation and histone deacetylation [5]. Downregulation of BNIP3 results in the failure of tumor cells to undergo cell death, and is associated with chemoresistance and poorer survival [6,7].

The transglutaminase 2 (TGM2) family catalyze formation of an amide bond between the carboxamide groups of peptide-bound glutamine residues and primary amino groups in various compounds [8]. One member of the TGM2 family, TGase 2 (TGM2) can enhance the survival of hypoxic cells and has been identified as a HIF-1α transcriptional target. Hypoxia upregulates *TGM2 *expression via a HIF-1α dependent pathway and concurrently activates intracellular TGM2 activity [9]. Increased expression of TGM2 is associated with drug resistance in cancer [10], due to activation of nuclear factor-kB (NF-kB) via cross-linking and polymerization of free I-kB by TGM2 [11].

Although BNIP3 and TGM2 are associated with drug resistance and provide valuable prognostic markers in a variety of cancers [6,7,10], the expression and significance of BNIP3 and TGM2 have not been investigated in patients with laryngeal SCC receiving postoperative radiotherapy. Therefore, we examined the pattern and correlation between TGM2 and BNIP3 expression to determine their association with clinical factors and outcome in patients with SCC of the larynx.

The results of this study indicate that, in addition to lymph node involvement, the expression levels of BNIP3 and TGM2 are novel independent predictive factors for survival in laryngeal SCC patients receiving postoperative radiotherapy.

## Methods

### Patients and tissue samples

This study was approved by the Institutional Review Board and Human Ethics Committee of Sun Yet-sen University Cancer Center. A total of 148 patients with histologically confirmed SCC of the larynx treated from 1997 to 2003 at the Sun Yet-sen University Cancer Center were included. Relevant clinical pathologic features (Table 1) were obtained from the medical files and/or by telephone interviews with the patient or their relatives. Tumor types and histological grade classifications were designated according to World Health Organization classification of Tumors: Pathology and Genetics of Head and Neck Tumors [12].

**Table 1:** Expression of TGM2 and BNIP3 and their relationship with clinicopathological characteristics in laryngeal squamous cell carcinoma patients

Features	No. of patients	TGM2		*P*	BNIP3		*P*
					
		High	Low		High	Low	
Gender							
Female	3(2.0%)	0	3	0.283^a^	1	2	0.194^b^
Male	145(98.0%)	70	75		105	40	
Age (years)^c^							
< 60	72(48.6%)	35	37	0.755	52	20	0.875
≥60	76(51.4%)	35	41		54	22	
Histological grade							
Well	70(47.3%)	37	33	0.184	50	20	0.933
Moderately	58(39.2%)	22	36		41	17	
Poorly	20(13.5%)	11	9		15	5	
Smoking index^d^							
< 600	67(45.3%)	36	31	0.154	52	15	0.651
≥600	81(54.7%)	34	47		54	27	
Alcohol consumption							
No	88(59.5%)	40	48	0.587	56	32	0.009**
Yes	60(40.5%)	30	30		50	10	
Primary Site							
Glottic	104(70.3%)	50	54	0.433	74	30	0.791
Supraglottic	39(26.3%)	19	20		29	10	
Subglottic	5(3.4%)	1	4		3	2	
T Stage							
1-2	101(68.2%)	47	54	0.785	71	30	0.600
3-4	47(31.8%)	23	24		35	12	
Cervical lymph node metastasis							
Positive	19(12.8%)	9	10	0.995	17	2	0.064
Negative	129(87.2%)	61	68		89	40	
TNM Stage							
I-II	96(64.9%)	46	50	0.838	67	29	0.502
III-IV	52(35.1%)	24	28		39	13	
Total radiation dose							
< 60 Gy	61(41.2%)	24	37	0.105	41	20	0.319
≥60 Gy	87(58.8%)	46	41		65	22	
Margin							
Negative	68(45.9%)	38	30	0.054	50	18	0.635
Positive	80(54.1%)	32	48		56	24	
BNIP3							
High	106(71.6%)	57	49	0.012*			
Low	42(28.4%)	13	29				

^a ^Continuity correction. ^b^Fisher's exact test. ^c ^Patients were divided according to the median age. ^d ^Smoking index is defined as the number of cigarettes used per day **× **the total smoking time (years). *P < 0.05, **P < 0.01

### Surgery

All patients underwent a major surgical intervention at Sun Yet-sen University Cancer Center; patients irradiated after excisional biopsies were not included in the study. In total, 116/148 (78.4%) of the laryngeal SCC patients were treated with partial laryngectomy and 32/148 (21.6%) were treated with total laryngectomy. Unilateral or bilateral neck node dissection was performed in 79 (53.4%) of the patients.

### Radiotherapy

The indications for postoperative radiotherapy in our hospital at the time of the study included clinically or microscopically positive surgical margins, pathologically confirmed positive neck nodes and/or the advanced clinical stages of primary laryngeal cancer (T3, T4). Only patients in good general condition with no distant metastases were considered for PRT.

The total radiation dose in the clinical target volume area ranged from 36 to 82 Gy, with a median value of 60 Gy. Lower doses (< 50 Gy) were given to patients whose clinical condition demanded premature termination of the treatment (due to local progression during PRT, deteriorating performance status or intercurrent disease) or patients who refused to complete treatment. Higher doses (> 70 Gy) were given to patients with a clinically palpable mass after surgery, or patients who required an increased total dose due to long duration treatment gaps.

### Immunohistochemistry (IHC) staining

**Figure 1:**
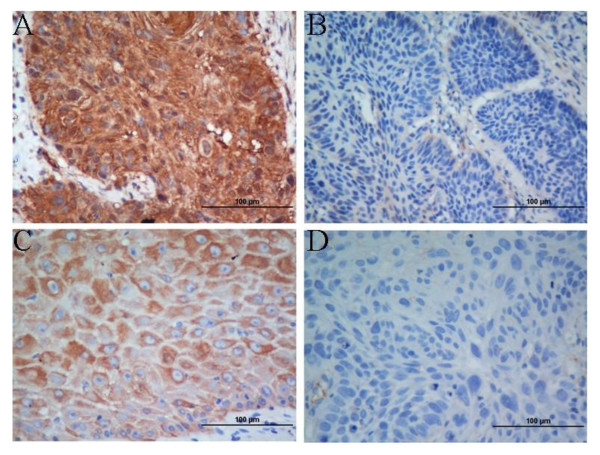
**Immunohistochemical staining for TGM2 and BNIP3 in laryngeal squamous cell carcinoma**. Positive expression of BNIP3 was mainly localized in the nucleus, but was also observed in the cytoplasm of tumor cells. Positive expression of TGM2 was mainly localized in the cytoplasm of tumor cells, but was also observed in the nucleus. Representative images of (A) highly positive BNIP3 expression in tumor cells, (B) low BNIP3 expression in tumor cells, (C) highly positive TGM2 expression in tumor cells and (D) low TGM2 expression in tumor cells (SP × 400)

A total of 148, routinely processed laryngeal squamous cell carcinoma paraffin-embedded samples were cut into 4 μm sections, dried overnight at 56°C, deparaffinized, rehydrated, antigen retrieval was performed in citrate buffer at 92°C using a microwave oven and the sections were incubated in 3% H_2_O_2 _to block endogenous peroxidase activity. The sections were incubated in mouse monoclonal [CUB 7402] anti-TGM2 antibody (ab2386, ABCAM, dilution 1:100) or mouse monoclonal [Ana40] anti-BNIP3 antibody (ab10433, ABCAM, dilution 1:100) overnight at 4°C in a humidified chamber, staining was visualized using an avidin-biotin technique and DAB staining, followed by hematoxylin nuclear counterstaining. Negative controls were prepared by omitting the primary antibody. Histological and IHC evaluation were performed independently by two pathologists while blinded to the clinicopathological outcomes of the patients. Slides with indeterminate evaluation were re-evaluated, and a consensus was reached. Briefly, each slide was examined in its entirety under a light microscope. Pilot studies indicated BNIP3 staining was both nuclear and cytoplasmic (Figure 1); therefore, the intensity of nuclear and cytoplasmic staining was defined as no staining (0), weak staining (1), moderate staining (2) or strong staining (3), and the percentage of cells stained was scored as 0-10% (1), 10-50% (2), 51-80% (3) or 81-100% (4). Tumors were considered highly positive if they demonstrated moderate or strong staining (2 or 3) in greater than 50% of cells (3 or 4). TGM2 expression was scored using the same intensity, percentage scores and positive criteria as BNIP3, as previously reported [13].

### Statistical methods

Statistical analysis was performed using SPSS 13.0 for Windows. The *χ^2 ^*test was used to evaluate categorical variables. Associations between clinicopathological features and TGM2 or BNIP3 immunohistochemical expression were analyzed using the Pearson chi-square test of independence. Multivariate survival analyses were performed using the Cox regression model. Overall survival (OS) was measured from the onset of treatment to the date of death or survival status at the last date of follow-up. OS probabilities were estimated using the Kaplan- Meier method and significant differences were assessed using the log-rank test. *P *values < 0.05 were considered statistically significant, and *P *values < 0.01 were considered strongly statistically significant.

## Results

### Clinicopathological features

In total, 148 laryngeal SCC patients (145 males and 3 females) with a median age of 60 years (range: 23-85 years) were included in this study. Table 1 presents a summary of the gender, age, tumor site, tumor stage, histological grade, smoking index, alcohol consumption, total radiation dose and surgery margins of the patients. According to the 6th Edition of the International Union Against Cancer (UICC) TNM classification system, 26 patients were Stage I, 70 were Stage II, 28 were Stage III and 24 were Stage IV.

### Patient outcomes after follow-up

**Figure 2:**
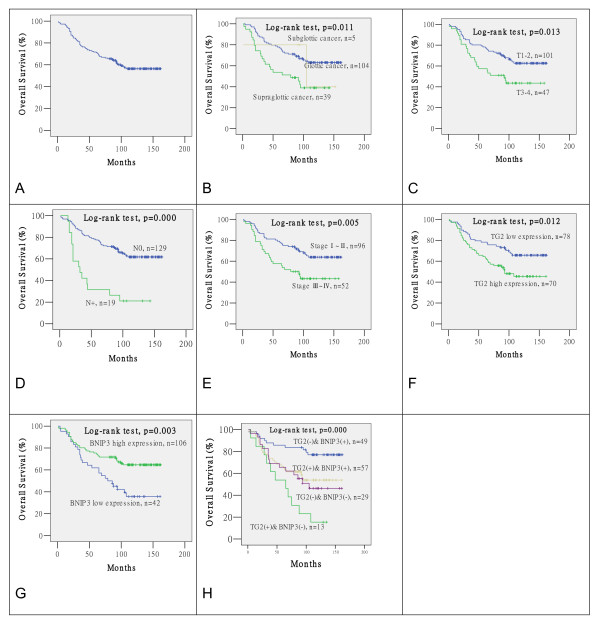
**Survival curves for laryngeal squamous cell carcinoma patients**. (A) Overall survival (OS) for all patients. (B) OS in patients with different primary tumor sites. (C) OS in patients with different T stages. (D) OS in patients with and without cervical lymph node metastasis. (E) OS in patients with different UICC stages. (F) OS in patients with high and low TGM2 expression. (G) OS in patients with high and low BNIP3 expression. (H) OS in patients with high and low BNIP3 and TGM2 expression.

The last follow-up date was June 29th, 2010, and no patients were lost during follow-up. The 3, 5 and 10 year overall survival rates (OS) for all patients were 77.7%, 71.6% and 56.4%, respectively (Figure 2). Sixty two cancer-related deaths were reported, with a median time to death of 35 months (range 1-108 months). There are 6 non-cancer-related deaths. The median time to non-cancer-related death is 30 months (range 18-78 months).

### Immunohistochemical expression of TGM2 and BNIP3

Positive expression of BNIP3 was mainly localized in the nucleus, with some expression detected in the cytoplasm of laryngeal SCC tumor cells. Positive expression of TGM2 was mainly localized in the cytoplasm of laryngeal SCC tumor cells, with some expression detected in the nucleus (Figure 1). The immunostaining scores are listed in the Table 1. TGM2 was highly positive in 47.3% (70/148) of the tumors, and BNIP3 was highly positive in 71.6% (106/148) of the tumors. Furthermore, highly positive BNIP3 expression was correlated with highly positive TGM2 expression (*P *= 0.012) and alcohol consumption (*P *= 0.009; Table 1).

### BNIP and TGM2 are associated with patient prognosis

Univariate analyses indicated a significant association between OS and primary tumor site, T stage, cervical lymph node metastasis, TNM stage, TGM2 expression and BNIP3 expression in laryngeal SCC (Table 2, Figure 2). Patients with both high BNIP3 expression and low TGM2 expression had a significantly better overall survival, compared to patients with low BNIP3 expression and high TGM2 expression, respectively (*P *< 0.001). The 3, 5 and 10-year overall survival rates (OS) of patients who expressed high levels of BNIP3 and low levels of TGM2 were 87.8%, 85.7% and 76.9%, respectively.

**Table 2:** The relationship of clinicopathological variables and immunohistochemical features with overall survival (OS) in laryngeal squamous cell carcinoma patients

Feature	No. of patients	Overall survival (%)			*χ^2^*	*P^a^*
				
		3 y	5 y	10 y		
Gender					1.539	0.215
Female	3(2.0%)	100	100	100		
Male	145(98.0%)	77.2	71.0	55.6		
Age (years)^b^					0.019	0.889
< 60	72(48.6%)	80.6	72.2	54.9		
≥60	76(51.4%)	75	71.1	57.6		
Histological grade					2.976	0.226
Well	70(47.3%)	85.7	81.4	60.8		
Moderately	58(39.2%)	69.0	62.1	51.2		
Poorly	20(13.5%)	75.0	65.0	55.0		
Smoking index^c^					3.745	0.053
< 600	67(45.3%)	83.6	77.6	66.2		
≥600	81(54.7%)	72.8	66.7	48.4		
Alcohol consumption					0.033	0.856
No	88(59.5%)	79.5	71.6	56.8		
Yes	60(40.5%)	75.0	71.7	55.6		
Primary Site					8.974	0.011*
Glottic	104(70.3%)	82.7	77.9	63.1		
Supraglottic	39(26.3%)	64.1	53.8	39.0		
Subglottic	5(3.4%)	80.0	80.0	40.0		
T Stage					6.201	0.013*
1-2	101(68.2%)	81.2	78.2	62.6		
3-4	47(31.8%)	70.2	57.4	43.5		
Cervical lymph node metastasis					21.263	< 0.001**
Negative	129(87.2%)	82.9	77.5	61.7		
Positive	19(12.8%)	42.1	31.6	21.1		
TNM Stage					7.736	0.005**
I-II	96(64.9%)	82.3	79.6	63.8		
III-IV	52(35.1%)	69.2	57.7	43.3		
Total radiation dose					1.671	0.196
< 60 Gy	61(41.2%)	78.7	75.4	62.9		
≥60 Gy	87(58.8%)	77.0	69.0	51.7		
Margin					0.395	0.530
Negative	68(45.9%)	82.4	76.5	57.9		
Positive	80(54.1%)	73.8	67.5	55.0		
TGM2					6.308	0.012*
High	70(47.3%)	72.9	64.3	45.5		
Low	78(52.7%)	82.1	78.2	65.7		
BNIP3					8.586	0.003**
High	106(71.6%)	79.2	75.5	64.4		
Low	42(28.4%)	71.4	61.9	35.7		
TGM2 & BNIP3					19.021	< 0.001**
TGM2 (-) & BNIP3 (+)	49(33.1%)	87.8	85.7	76.9		
TGM2 (+) & BNIP3 (-)	13(8.8%)	69.2	53.8	15.4		
TGM2 (+) & BNIP3 (+)	57(38.5%)	73.7	66.7	53.8		
TGM2 (-) & BNIP3 (-)	29(19.6%)	72.4	65.5	46.3		

^a ^Log-rank test. ^b ^Patients were divided into two groups according to the median age. ^c ^Smoking index is defined as the number of cigarettes used per day **× **the total smoking time (years). *P < 0.05, **P < 0.01

### Multivariate analysis

Multivariate survival analysis was performed using the Cox regression model to calculate the odds ratio and 95% confidence intervals for each clinicopathological variable. The model was simplified in a stepwise fashion by removing variables with a *P *value ≥0.05. The analysis revealed that TGM2 expression status, BNIP3 expression status and cervical lymph node metastasis were statistically significant independent predictive factors for OS in laryngeal SCC patients (Table 3).

**Table 3:** Multivariate analysis of the effect of clinicopathological factors on overall survival in laryngeal squamous cell carcinoma patients

Variables	Overall survival		
	
	Odds ratio	95% CI	*P *values
Cervical lymph node metastasis	3.708	1.952-7.041	< 0.001**
TGM2	2. 899	1.669-5.036	< 0.001**
BNIP3	0.324	0.188-0.559	< 0.001**
Primary Site	1.460	0.934-2.283	0.097
T Stage	1.485	0.871-2.534	0.147

*P < 0.05, **P < 0.01

## Discussion

In this study we evaluated the significance of various prognostic factors in patients with SCC of the larynx treated with primary surgery and PRT. No patients received any other form of adjuvant systemic treatment. The overall survival (OS) of the patients was significantly related to the UICC stage, in agreement with previous reports that the initial classification (T stage, N stage and total stage) are significant prognostic factors for survival in laryngeal SCC [14-17]. In this study, univariate analysis also indicated that tumor sites in the supraglottis were correlated with a significantly poorer prognosis.

It has been reported that the number, site and volume of metastatic lymph nodes can negatively influence the prognosis in laryngeal carcinoma [18,19], and these results were confirmed by our study, as univariate analysis indicated that the presence of metastatic lymph nodes (*P *< 0.001) had a negative prognostic value. The relationship between nodal involvement and local control has been examined in several patient series. van den Bogaert et al. and Wall et al. [20,21] reported 5 year local control rates of 70% and 79% in patients without positive node involvement, compared to 49% and 57% in patients with clinically positive nodes. Three large multicenter studies of laryngeal SCC patients treated with PRT have reported similar recurrence rates [22-24], indicating that cervical lymph node status is the most important tumor-related prognostic factor in head and neck cancer patients. Additionally, the incidence of local recurrence and risk of distant metastases increase as the tumor burden in the neck increases [25,16].

To our knowledge, no previous studies have evaluated the prognostic value of TGM2 and BNIP3 in head-and-neck cancer in patients treated with PRT. In this study, univariate analyses and multivariate analysis indicated that high expression of TGM2 and/or low expression of BNIP3 are associated with poorer overall survival in laryngeal SCC patients receiving PRT.

Several factors may explain the association between high expression of TGM2 and poorer OS. TGM2 expression can promote cell surface interaction with fibronectin and protect breast cancer cells from apoptosis [26]. Intense expression at the stromal-epithelial interface suggests that TGM2 plays a role in cell adhesion, cell migration, invasiveness and metastasis [27], and Dardik et al. reported that stromal expression of TGM2 in the endothelium of newly formed blood vessels may lead to tumor growth and metastasis [28]. Additionally, TGM2 can activate NF-κB by crosslinking and polymerization, or by destabilizing the association of the p65/p50 (NF-κB) complex with IκB [27]. Aberrant activation of NF-κB complexes can contribute to tumorigenesis by regulating expression of genes which promote cancer cell growth and survival. Constitutive expression of NF-κB has also been implicated in tumor drug resistance. Recent experimental studies by Dae-Seok Kim et al. demonstrated that inhibition of TGase 2 using siRNA, cystamine, glucosamine or R2 peptides promotes cell death in drug-resistant cancer cells via NF-κB inactivation [29]; However, Barnes et al. [30] suggested that reduced TGM2 activity is associated with tumor growth and metastasis, while Fesus et al. reported that increased activity of TGM2 in cells undergoing apoptosis indicates a pro-apoptotic function [31]. Collectively, these studies indicate that the role of TGM2 may depend on the type of cancer, the cell type and cancer stage; and this study demonstrates that high positive expression of TGM2 is associated with poorer overall survival in laryngeal SCC patients receiving surgery and postoperative radiotherapy.

Little is known about how TGM2 expression may affect the treatment efficacy of radiotherapy. Yuan et al. reported that the small molecule TGM2 inhibitor KCC009, which inhibits the ability of TGM2 to bind fibronectin and prevents the disposition of linear fibronectin strands in the ECM, promotes apoptosis and enhances radiosensitivity in cultured IOMM-Lee meningioma cells and meningioma tumor explants [32]. Mian et al. discovered that overexpression of TGM2 or a cross-linking defective TGM2 mutant (C277S) in malignant hamster fibrosarcoma cells (MetB) lead to delayed S-phase to G2/M progression [33]. As the G2/M-phase is the most sensitive phase to radiation, this observation may explain the association between overexpression of TGM2 and adverse outcome in patients receiving radiotherapy. Though this evidence strongly suggests a role for TGM2 in cancer, the exact molecular mechanism which by high levels of TGM2 expression lead to an adverse prognosis in laryngeal SCC is not know and requires further research.

BNIP3 is strongly upregulated in response to hypoxia. Generally, overexpression of BNIP3 induces necrotic-like cell death, autophagy or apoptotic cell death. However, it has been suggested that the ability of BNIP3 to induce cell death is blocked in cancer cells [34]. BNIP3 is downregulated in pancreatic ductal adenocarcinoma and correlates with reduced patient survival [5,6]. *BNIP3 *is also downregulated in oxaliplatin-resistant colon cancer cells [7] and knockdown of BNIP3 using siRNA or S100A4 results in resistance to fluorouracil (5-FU) and/or gemcitabine in pancreatic ductal adenocarcinoma cells [6], indicating that BNIP3 is an important regulator during drug-induced cell death in cancer cells.

This study indicates that reduced expression of BNIP3 is associated with poorer overall survival in laryngeal SCC patients receiving PRT. The ability of BNIP3 to affect the efficacy of radiotherapy is poorly characterized. Kennedy et al. observed that overexpression of the apoptosis-related gene BNIP3 could affect the sensitivity of CRC cells to combined paclitaxel, radiation, and 5-FU therapy [35], and the molecular mechanism by which BNIP3 expression affects the prognosis of patients with laryngeal SCC requires further study.

In this study we observed that BNIP3 expression was correlated with TGM2 expression in laryngeal SCC (*P *= 0.012). TGM2 activates NF-κB, via crosslinking and polymerization or destabilizing the association of the p65/p50 (NF-κB) complex with IκB [27]. Therefore, we hypothesize that increased expression of TGM2 leads to down-regulation of BNIP3 because BNIP3 expression can be repressed through the transcription factor nuclear factor kappa B (NF-κB) binding to the bnip3 promoter [36].

The results of this study should be interpreted with caution as this is a retrospective study of a relatively small number of samples. Use of alternative methodologies such as microdissection and in-situ hybridization and/or RT-PCR are required to confirm these results at the RNA level.

## Conclusions

The results of this study indicate that the expression levels of BNIP3 and TGM2 are novel predictive factors of survival in laryngeal SCC patients treated with surgery and postoperative radiotherapy, and further study is required to determine the molecular mechanisms by which BNIP3 and TGM2 influence the prognosis of these patients. Additionally, the mechanism regulating the correlation between TGM2 and BNIP3 expression in laryngeal SCC requires further investigation.

## Abbreviations

PRT: Postoperative radiotherapy; SCC: Squamous cell carcinoma; HIF-1: Hypoxia-inducible factor-1; TGM2: Transglutaminase 2; NF-kB: Nuclear factor-kB; IHC: Immunohistochemistry; UICC: International Union Against Cancer; OS: Overall survival.

## Competing interests

The authors declare that they have no competing interests.

## Authors' contributions

TJ, HXL, LBG, HL, RS, WKC, and QLL carried out the cases collection, TJ and HL carried out the immunohistochemical staining work, TJ, HL, XYC and WHH analyzed results. WHH conceived of the study, participated in its design and coordination and helped to draft the manuscript. All authors read and approved the final manuscript.

## References

[B1] Jemal A Siegel R Ward E Hao Y Xu J Murray T Thun MJ Cancer statistics CA Cancer J Clin 2008 58 71 96 10.3322/CA.2007.0010 18287387

[B2] Bruick RK Expression of the gene encoding the proapoptotic Nip3 protein is induced by hypoxia Proc Natl Acad Sci USA 2000 97 9082 9087 10.1073/pnas.97.16.9082 10922063 PMC16825

[B3] Guo K Searfoss G Krolikowski D Pagnoni M Franks C Clark K Yu KT Jaye M Ivashchenko Y Hypoxia induces the expression of the pro-apoptotic gene BNIP3 Cell Death Differ 2001 8 367 376 10.1038/sj.cdd.4400810 11550088

[B4] Regula KM Ens K Kirshenbaum LA Inducible expression of BNIP3 provokes mitochondrial defects and hypoxia-mediated cell death of ventricular myocytes Circ Res 2002 91 226 231 10.1161/01.RES.0000029232.42227.16 12169648

[B5] Okami J Simeone DM Logsdon CD Silencing of the hypoxia-inducible cell death protein BNIP3 in pancreatic cancer Cancer Res 2004 64 5338 5346 10.1158/0008-5472.CAN-04-0089 15289340

[B6] Erkan M Kleeff J Esposito I Giese T Ketterer K Büchler MW Giese NA Friess H Loss of BNIP3 expression is a late event in pancreatic cancer contributing to chemoresistance and worsened prognosis Oncogene 2005 24 4421 4432 10.1038/sj.onc.1208642 15856026

[B7] Tang H Liu YJ Liu M Li X Establishment and gene analysis of an oxaliplatin-resistant colon cancer cell line THC8307/L-OHP Anticancer Drugs 2007 18 633 639 10.1097/CAD.0b013e3280200428 17762391

[B8] Folk JE Transglutaminases Annu Rev Biochem 1980 49 517 531 10.1146/annurev.bi.49.070180.002505 6105840

[B9] Jang GY Jeon JH Cho SY Shin DM Kim CW Jeong EM Bae HC Kim TW Lee SH Choi Y Lee DS Park SC Kim IG Transglutaminase 2 suppresses apoptosis by modulating caspase 3 and NF-kappaB activity in hypoxic tumor cells Oncogene 2010 29 356 367 10.1038/onc.2009.342 19838207

[B10] Mehta K Fok J Miller FR Koul D Sahin AA Prognostic significance of tissue transglutaminase in drug resistant and metastatic breast cancer Clin Cancer Res 2004 10 8068 8076 10.1158/1078-0432.CCR-04-1107 15585642

[B11] Lee J Kim YS Choi DH Bang MS Han TR Joh TH Kim SY Transglutaminase 2 induces nuclear factor-kappaB activation via a novel pathway in BV-2 microglia J Biol Chem 2004 279 53725 53735 10.1074/jbc.M407627200 15471861

[B12] Thompson L World Health Organization classification of tumours: pathology and genetics of head and neck tumours Ear Nose Throat J 2006 85 74 16579185

[B13] Boddy JL Fox SB Han C Campo L Turley H Kanga S Malone PR Harris AL The androgen receptor is significantly associated with vascular endothelial growth factor and hypoxia sensing via hypoxia-inducible factors HIF-1a, HIF-2a, and the prolyl hydroxylases in human prostate cancer Clin Cancer Res 2005 11 7658 7663 10.1158/1078-0432.CCR-05-0460 16278385

[B14] Bastit L Blot E Debourdeau P Menard J Bastit P Le Fur R Influence of the delay of adjuvant postoperative radiation therapy on relapse and survival in oropharyngeal and hypopharyngeal cancers Int J Radiat Oncol Biol Phys 2001 49 1 139 146 10.1016/S0360-3016(00)01376-6 11163507

[B15] Leemans CR Tiwari R Nauta JJ van der Waal I Snow GB Recurrence at the primary site in head and neck cancer and the significance of neck node metastases as a prognostic factor Cancer 1993 73 1 187 190 8275423 10.1002/1097-0142(19940101)73:1<187::aid-cncr2820730132>3.0.co;2-j

[B16] Leibel SA Scott CB Mohiuddin M Marcial VA Coia LR Davis LW Fuks Z The effect of local-regional control on distant metastatic dissemination in carcinoma of the head and neck: results of an analysis from the RTOG head and neck database Int J Radiat Oncol Biol Phys 1991 21 3 549 556 10.1016/0360-3016(91)90669-U 1869453

[B17] Richard JM Sancho-Garnier H Micheau C Saravane D Cachin Y Prognostic factors in cervical lymph node metastasis in upper respiratory and digestive tract carcinomas: study of 1713 cases during a 15-year period Laryngoscope 1987 97 1 97 101 3796181 10.1288/00005537-198701000-00019

[B18] Stell PM Prognosis in laryngeal carcinoma: tumor factors Clin Otolaryngol 1990 15 1 69 81 10.1111/j.1365-2273.1990.tb00436.x 2323084

[B19] Hoffstetter S Malissard L N'Guyen TD Panis X Jung GM Bachaud JM Prevost B Quint R Chaplain G Eschwège F Rambert P Fleury-Touzeau F Results of postoperative cervical node irradiation in carcinoma of the pharyngo-larynx. A study of the cooperative group of radiotherapist Bull Cancer Radiother 1996 83 2 86 89 8688224

[B20] van den Bogaert W Ostyn F van der Schueren E The different clinical presentation, behaviour and prognosis of carcinomas originating in the epilarynx and lower supraglottis Radiother Oncol 1983 1 2 117 131 10.1016/S0167-8140(83)80015-2 6680217

[B21] Wall TJ Peters LJ Brown BW Oswald MJ Milas L Relationship between lymph nodal status and primary tumor control probability in tumors of the supraglottic larynx Int J Radiat Oncol Biol Phys 1985 11 11 1895 1902 10.1016/0360-3016(85)90269-X 4055449

[B22] Kramer S Gelber RD Snow JB Marcial VA Lowry LD Davis LW Chandler R Combined radiation therapy and surgery in the management of advanced head and neck cancer: final report of the study 73-03 of the Radiation Therapy Oncology Group Head Neck Surg 1987 10 1 19 30 10.1002/hed.2890100105 3449477

[B23] Nguyen TD Malissard L Théobald S Eschwège F Panis X Bachaud JM Rambert P Chaplain G Quint R Advanced carcinoma of the larynx: results of surgery and radiotherapy without induction chemotherapy (1980-1985): a multivariate analysis Int J Radiat Oncol Biol Phys 1996 5 5 1013 1018 8985021 10.1016/s0360-3016(96)00355-0

[B24] Wolf GT Hong WK Fisher SG Induction chemotherapy plus radiation compared with surgery plus radiation in patients with laryngeal cancer N Engl J Med 1991 324 24 1685 1690 2034244 10.1056/NEJM199106133242402

[B25] Leibel SA Fuks Z Beck-B ornholdt HP The impact of local tumor control on the outcome of human cancer Current topics in clinical radiobiology of tumors 1993 Berlin: Springer-Ve rlag 113 127

[B26] Mangala LS Fok JY Zorrilla-Calancha IR Verma A Mehta K Tissue transglutaminase expression promotes cell attachment, invasion and survival in breast cancer cells Oncogene 2007 26 2459 2470 10.1038/sj.onc.1210035 17043648

[B27] Mann AP Verma A Sethi G Manavathi B Wang H Fok JY Kunnumakkara AB Kumar R Aggarwal BB Mehta K Overexpression of tissue transglutaminase leads to constitutive activation of nuclear factor-kappaB in cancer cells: delineation of a novel pathway Cancer Res 2006 66 8788 8795 10.1158/0008-5472.CAN-06-1457 16951195

[B28] Dardik R Inbal A Complex formation between tissue transglutaminase II (tTG) and vascular endothelial growth factor receptor 2 (VEGFR-2): proposed mechanism for modulation of endothelial cell response to VEGF Exp Cell Res 2006 312 2973 2982 10.1016/j.yexcr.2006.05.019 16914140

[B29] Kim DS Park KS Jeong KC Lee BI Lee CH Kim SY Glucosamine is an effective chemo-sensitizer via transglutaminase 2 inhibition Cancer Lett 2009 273 243 249 10.1016/j.canlet.2008.08.015 18804908

[B30] Barnes RN Bungay PJ Elliott BM Walton PL Griffin M Alterations in the distribution and activity of transglutaminase during tumour growth and metastasis Carcinogenesis 1985 6 459 463 10.1093/carcin/6.3.459 2858274

[B31] Fesus L Thomazy V Falus A Induction and activation of tissue transglutaminase during programmed cell death FEBS Lett 1987 224 104 108 10.1016/0014-5793(87)80430-1 2890537

[B32] Yuan L Behdad A Siegel M Khosla C Higashikubo R Rich KM Tissue transgluaminase 2 expression in meningiomas J Neurooncol 2008 90 125 132 10.1007/s11060-008-9642-1 18587533 PMC3732188

[B33] Mian S el Alaoui S, Lawry J, Gentile V, Davies PJ, Griffin M: The importance of the GTP-binding protein tissue transglutaminase in the regulation of cell cycle progression FEBS Lett 1995 370 27 31 10.1016/0014-5793(95)00782-5 7649299

[B34] Kothari S Cizeau J McMillan-Ward E Israels SJ Bailes M Ens K Kirshenbaum LA Gibson SB BNIP3 plays a role in hypoxic cell death in human epithelial cells that is inhibited by growth factors EGF and IGF Oncogene 2003 22 4734 4744 10.1038/sj.onc.1206666 12879018

[B35] Kennedy AS Harrison GH Mansfield CM Zhou XJ Xu JF Balcer-Kubiczek EK Survival of colorectal cancer cell lines treated with paclitaxel, radiation, and 5-FU: effect of TP53 or hMLH1 deficiency Int J Cancer 2000 90 175 185 10.1002/1097-0215(20000820)90:4<175::AID-IJC1>3.0.CO;2-W 10993958

[B36] Baetz D Regula KM Ens K Shaw J Kothari S Yurkova N Kirshenbaum LA Nuclear factor-kappaB-mediated cell survival involves transcriptional silencing of the mitochondrial death gene BNIP3 in ventricular myocytes Circulation 2005 112 3777 3785 10.1161/CIRCULATIONAHA.105.573899 16344406

